# Risk Factors and Effect of Intrathoracic Anastomotic Leakage after Esophagectomy for Underlying Malignancy—A Ten-Year Analysis at a Tertiary University Centre

**DOI:** 10.3390/clinpract12050081

**Published:** 2022-09-26

**Authors:** Nader El-Sourani, Sorin Miftode, Fadl Alfarawan, Achim Troja, Maximilian Bockhorn

**Affiliations:** Department for General and Visceral Surgery, University Hospital Oldenburg, Klinikum Oldenburg AöR, 26133 Oldenburg, Germany

**Keywords:** esophagectomy, intrathoracic anastomosis, anastomotic leakage, diabetes, risk factors

## Abstract

Aim: Surgical resection remains the treatment of choice for curable esophageal cancer patients. Anastomotic leakage after esophagectomy with an intrathoracic anastomosis is the most feared complication, and is the main cause of postoperative morbidity and mortality. The aim of this study was to identify risk factors associated with anastomotic leakage and its effect on the postoperative outcome. Methods: Between 2012 and 2022, all patients who underwent Ivor Lewis esophagectomy for underlying malignancy were included in this study. We performed a retrospective analysis of 174 patients. The dataset was analyzed to identify risk factors for the occurrence of anastomotic leakage. Results: A total of 174 patients were evaluated. The overall anastomotic leakage rate was 18.96%. The 30-day mortality rate was 8.62%. Multivariate logistic regression analysis identified diabetes (*p* = 0.0020) and obesity (*p* = 0.027) as independent risk factors associated with anastomotic leakage. AL had a drastic effect on the combined ICU/IMC and overall hospital stay (*p* < 0.001. Conclusion: Anastomotic leakage after esophagectomy with intrathoracic anastomosis is the most feared complication and major cause of morbidity and mortality. Identifying risk factors preoperatively can contribute to better patient management.

## 1. Introduction

Esophageal carcinoma is one of the most rapidly increasing tumor entities in the western world. Despite remarkable progress in the treatment of patients with esophageal cancer, the overall outcome is still limited. The five- and ten-year survival rates are 35% and 17%, respectively [1]. In addition, post-operative morbidity is extremely high. The esophageal complications consensus group (ECCG) has shown that a total of 1046 of 1617 (65%) patients had a postoperative complication, including 468 (29%) patients with a major complication [2]. The most common feared complication is anastomotic leakage, which was seen in 19% of the patients. Despite improvements in surgical technique and perioperative management, the overall mortality rate across low, intermediate and high-volume centers is 7.7%, and reaches up to 50% in patients with postoperative anastomotic leakage [3]. Several factors can be attributed to an increased risk of developing anastomotic leakage. They can be grouped into preoperative, intraoperative and postoperative risk factors. Intraoperative risk factors include surgical-related techniques such as the surgical approach (open vs. laparoscopic), the location of the anastomosis (intrathoracic vs. cervical) and the type of conduit (gastric vs. intestinal) [4,5]. To date, most of the available studies have compared intrathoracic versus cervical anastomosis; however, there are significant differences in the clinical manifestation, severity, prognosis and incidence between intrathoracic and cervical anastomotic leakage [6,7]. Therefore, there is a lack of data concentrating on risk factors of anastomotic leakage after esophagectomy with an intrathoracic anastomosis.

The objective of the study was to investigate the risk factors associated with the presence of an intrathoracic anastomotic leakage, as well its effect on the postoperative outcome of the patients.

## 2. Material and Methods

### 2.1. Study Population

A total of 174 patients underwent Ivor Lewis esophagectomy with intrathoracic anastomosis for underlying curable esophageal cancer at our department for General- and Visceral Surgery between 2012 and 2022. All patients, including those who developed post-surgical anastomotic leakage, were further analyzed. The following parameters were examined: comorbidities classified as cardiac, pulmonary, diabetic (type I and II)) and smoking, obesity (defined as a BMI greater than 25), combined IMC/ICU and overall hospital stay, tumor location, tumor histology, ASA classification, neoadjuvant therapy, number of lymph nodes harvested, operation method, morbidity and mortality

### 2.2. Surgical Method

All patients were positioned in the left-lateral prone position and underwent a standard operative Ivor Lewis esophagectomy with a 2-field lymphadenectomy. The abdominal part was performed either via a conventional horizontal laparotomy or laparoscopically. The thoracic part was performed through a muscle-preserving right thoracotomy. The anastomosis was performed using a circular stapler in all patients. Hybrid minimally invasive esophagectomy (HMIE) was recently introduced as a first step and is preferred over totally minimally invasive esophagectomy (TMIE), as it seems to be associated with a lower rate of anastomotic leakage [8,9].

### 2.3. Anastomotic Leakage

Anastomotic leakage was defined as the passage of intraluminal content to an extraluminal space through a defect in the continuity of the intestinal wall at the site of the anastomosis. Diagnosing an anastomotic leakage at our tertiary center was achieved through computed tomography (CT), as well as an upper endoscopy (UE). 

### 2.4. Statistical Analysis

Statistical analysis was performed using IBM SPSS Statistics Version 24 64-Bit-Version for Mac OS (IBM CO., Armonk, NY, USA). Continuous variables were presented as medians. To compare the variables, we employed a univariate and multivariate linear regression model. Categorical variables were compared using the chi-squared test. A Mann–Whitney U-test was performed to discover the effect of leakage on the length of combined IMC/ICU and overall hospital stay. Statistical significance was defined as *p* = 0.05.

## 3. Results

A total of 174 patients underwent Ivor Lewis esophagectomy for underlying malignancy in our surgical department between 2012 and 2022. All relevant patient characteristics are presented in Table 1. In total, 113 (80.14%) patients were male. A total of 33 (18.96%) patients developed an anastomotic leak after Ivor Lewis esophagectomy. Of the 33 patients, 30 were male and 3 were female. The median age was 61 years (range: 41–81 years) for the no leakage group and 58.5 years (range: 32–38 years) for the leakage group. The median American Society of Anesthesiologists classification (ASA) was 2, and 15 (8.62%) out of 174 patients died. The mortality rate within the no leakage group and the leakage group was 4.25% and 27.27%, respectively. Overall, adenocarcinoma was the most dominant histological diagnosis (n = 152, 89.08%), with the distal third of the esophagus being the most dominant location (n = 148, 85.05%)

The baseline characteristics comparing the groups are shown in Table 1. Diabetes and obesity, as well as surgical procedure and tumor location, were associated with a higher rate of anastomotic leakage (*p* < 0.05). Gender, histological type, neoadjuvant therapy, ASA classification and extension of lymph node harvest were not associated with anastomotic leakage.

Table 2 shows the results of multivariate logistic regression analysis, revealing diabetes, obesity and surgical procedure as statistically significant factors associated with anastomotic leakage (*p* < 0.05).

Table 3 depicts the effect of intrathoracic leakage on the combined IMC/ICU stay and overall hospital stay. The median IMC/ICU and median overall hospital stay in the no leakage group was 5.7 days and 21.3 days, respectively. The median IMC/ICU and median overall hospital stay in the leakage group was 33.3 days and 62.4 days, respectively. Therefore, anastomotic leakage had a drastic effect on the overall IMC/ICU, as well as the overall hospital stay (*p* < 0.001).

## 4. Discussion

Anastomotic leakage is the most feared complication after Ivor Lewis esophagectomy for underlying malignancy, and it is associated with higher morbidity, mortality, prolonged intensive care unit and hospital stay, which subsequently lead to increased hospital costs and negative long-term outcomes, such as worse long-term survival and quality of life [10,11]. Compared to China, adenocarcinoma is the dominant histological type in the western part of the world, and therefore risk factors associated with an anastomotic intrathoracic leakage may differ [12].

Factors resulting in poor tissue perfusion are often associated with an increased risk of developing an anastomotic insufficiency [13,14,15,16]. Gastric ischemic preconditioning was considered to reduce the incidence and severity of anastomotic leaks; however, multiple meta-analyses have proven the contrary [17,18]. Gooszen et al. have shown that diabetes, ASA grades of III and IV and COPD were identified as independent risk factors for the development of anastomotic leakage, with diabetes being the most statistically significant (*p* < 0.05) [19,20,21]. The same was true for our cohort, as we identified diabetes (*p* < 0.05) and obesity (*p* < 0.05) as independent risk factors associated with an anastomotic leakage. In addition, Kassis et al. analyzed 7595 esophagectomies, with 804 leaks, and have shown that factors associated with anastomotic leakage included diabetes and obesity [14]. Furthermore, our results have shown that surgical procedure is associated with a higher risk of anastomotic leakage (*p* < 0.05). This should be taken with a grain of salt, since we just recently shifted to a hybrid procedure, and therefore, most cases with anastomotic leakage are associated with an open approach. However, several studies have shown the benefits of a hybrid vs. an open approach, as it was associated with decreased morbidity, decreased blood loss and an overall better quality of life [22,23]. In addition, HMIE is currently favored over TMIE at our institution, as TMIE is associated with a higher rate of anastomotic leakage despite having moderately lower morbidity rates. However, a randomized-controlled study comparing HMIE versus TMIE is missing [8,9]. Moreover, the length of stay was higher for patients with anastomotic leakage, which is in accordance with our results. The combined IMC/ICU stay increased by nearly sevenfold, while the overall hospital stay tripled (*p* < 0.001). To our knowledge, our study is one of few that identified obesity as a predisposing factor. Our findings suggest that it is of utmost importance to improve the preoperative status of high-risk patients undergoing elective esophagectomy for underlying malignancy. Cardiac and pulmonary factors, as well as neoadjuvant therapy, could not be identified as predictors for anastomotic leakage. Unfortunately, details regarding the type of chemotherapy given, as well as the amount of radiation delivered, were not collected in this database. This might provide a level of ambiguity regarding the true safety of neoadjuvant treatment as an adjunct to surgical resection. Furthermore, our results have shown that anastomotic leakage has a drastic effect on the combined intensive care and overall hospital stay. Recent data suggest that age and comorbidity are risk factors for a prolonged hospital stay in patients who suffered an anastomotic leakage [10].

## 5. Limits of the Study

The limitations of this study are its retrospective design and lack of randomization. However, the strengths of this study are the relatively large sample size and the fact that all patients were operated on by the same two surgeons.

## 6. Conclusions

In summary, our results suggest that diabetes and obesity are independent risk factors associated with the occurrence of an anastomotic leakage. In addition, anastomotic leakage has a drastic effect on the postoperative period of the patient, increasing the time spent in the intensive care unit and the overall hospital stay by at least threefold.

## Figures and Tables

**Table 1:** Clinical and pathological characteristics in correlation with intrathoracic anastomotic leakage.

Variables	No Leakage	Leakage	*p*-Value ^a^
**Age (years, median, range)**	61 (range: 41–81)	58.5 (range: 32–83)	
**Male, n (%)**	113 (80.14%)	29 (87.87%)	0.221
**Comorbidity, n (%)**			
Cardiac	81 (56.25%)	19 (57.57%)	0.574
Pulmonary	25 (17.73%)	6 (18.18%)	0.563
Diabetes	11 (7.80%)	8 (24.24%)	0.012
Smoking	52 (36.87%)	13 (39.39%)	0.468
Obesity			0.033
**Tumor location, n (%)**			
Proximal	2 (1.38%)	0 (0%)	0.656
Middle	22 (15.60%)	1 (3.03 %)	0.040
Distal	116 (82.26%)	32 (96.96%)	0.022
**Surgical Procedure, n (%)**			
Open	96 (68.08%)	28 (84.84%)	0.040
Laparoscopic	45 (31.91%)	5 (15.15%)	0.040
**Lymph Node Harvest, n (%)**			
20 or less	58 (41.13%)	18 (54.54%)	0.115
21 or more	83 (58.86%)	15 (45.45%)	0.115
**Histological Type, n (%)**			
Adenocarcinoma	122 (86.52%)	30 (90.90%)	0.364
Squamous cell carcinoma	19 (13.47%)	3 (9.09%)	0.364
**Neoadjuvant therapy, n (%)**			
Chemotherapy	51 (36.17%)	16 (48.48%)	0.134
Radiochemotherapy	39 (27.65%)	5 (15.15%)	0.100
**ASA ^b^ Classification, n (%)**			
ASA 1	1 (0.69%)	1 (3.03)	0.344
ASA 2	67 (47.51%)	18 (54.54%)	0.297
ASA 3	68 (48.22%)	14 (42.42%)	0.343
ASA 4	5 (3.54%)	0 (0%)	0.345

^a^ Calculated via chi-square-test. ^b^ American Society of Anesthesiologists.

**Table 2:** Factors associated with occurrence of anastomotic leakage.

Variable	OR	95% CI	*p*-Value ^a^
Obesity	3.558	1.153–10.983	0.027
Diabetes	4.241	1.260–14.2777	0.020
Open/Laparoscopic	4.378	1.327–14.443	0.015

^a^ Calculated via multivariate logistic regression analysis.

**Table 3:** Effects of intrathoracic anastomotic leakage on combined IMCU/ICU and overall hospital stay in days.

	No Leakage	Leakage	*p*-Value ^a^
Combined IMC/ICU ^b^ stay (days, median)	5.7	33.3	<0.001
Overall hospital stay (days, median)	21.3	62.4	<0.001

^a^ Calculated via Mann–Whitney U test. ^b^ Intermediate Care/Intensive Care Unit.

## Data Availability

The data presented in this study are available on request from the corresponding author. The data are not publicly available due to data management of the hospital.
